# 6-(2-Chloro­benz­yl)-1-(4-chloro­phen­yl)-7-hy­droxy-2,3-dihydro-1*H*-imidazo[1,2-*a*]pyrimidin-5-one

**DOI:** 10.1107/S160053681003919X

**Published:** 2010-10-09

**Authors:** Waldemar Wysocki, Dariusz Matosiuk, Marzena Rządkowska, Zbigniew Karczmarzyk, Zofia Urbańczyk-Lipkowska, Przemysław Kalicki

**Affiliations:** a Department of Chemistry, University of Podlasie, ul. 3 Maja 54, 08-110 Siedlce, Poland; b Department of Synthesis and Chemical Technology of Pharmaceutical Substances, Medical University, ul. Staszica 6, 20-081 Lublin, Poland; c Institute of Organic Chemistry, Polish Academy of Sciences, ul. Kasprzaka 44/52, 01-224 Warsaw 42, POB 58, Poland

## Abstract

The title compound, C_19_H_15_Cl_2_N_3_O_2_, was obtained by a one-step cyclo­condensation of 2-amino-1-(4-chloro­phen­yl)imidazoline with diethyl (2-chloro­benz­yl)malonate under basic conditions. In the crystalline state, the mol­ecule exists as the 7-hy­droxy-5-oxo tautomer. The dihedral angles between the fused imidazopyrimidine and aromatic chloro­phenyl and chloro­benzyl rings are 14.2 (1) and 70.7 (1)°, respectively. The conformation of the mol­ecule is influenced by the intra­molecular C—H⋯O and C—H⋯N hydrogen bonds, giving a nearly planar five-ring fused system [maximum deviation from the mean plane = 0.296 (2) Å]. In the crystal structure, strong inter­molecular O—H⋯O hydrogen bonds link the mol­ecules into chains along the *c* axis. These chains are further stabilized by weak C—H⋯Cl and π–π inter­actions [centroid–centroid distance = 3.6707 (12) Å].

## Related literature

For background to dioxo derivatives of fused imidazoline ring systems, their biological activity and medical applications, see: Matosiuk, Fidecka, Antkiewicz-Michaluk, Dybała *et al.* (2002) ▶; Matosiuk, Fidecka, Antkiewicz-Michaluk, Lipkowski *et al.* (2002 ▶). For the synthesis, see: Rządkowska *et al.* (2004 ▶). For a related structure, see: Wysocki *et al.* (2006 ▶). For structure inter­pretation tools, see: Allen *et al.* (1995 ▶); Allen (2002 ▶); Bruno *et al.* (2002 ▶). For resonance-assisted hydrogen bonds, see: Gilli *et al.* (1989 ▶).
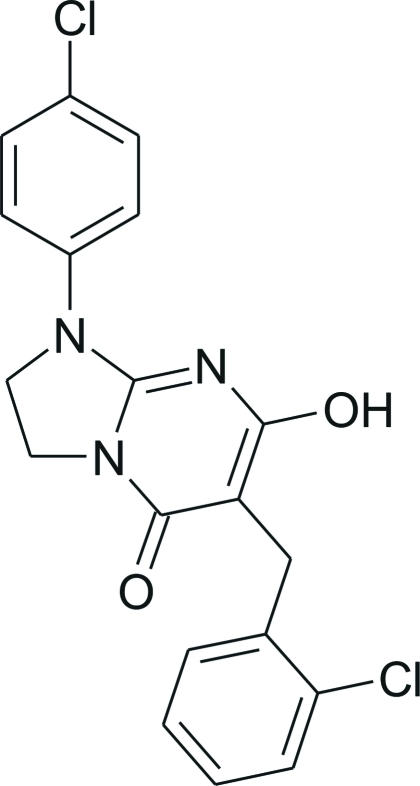


## Experimental

### 

#### Crystal data

C_19_H_15_Cl_2_N_3_O_2_
*M* *_r_* = 388.24
Monoclinic, 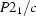

*a* = 11.4521 (3) Å
*b* = 12.8287 (4) Å
*c* = 11.7255 (3) Å
β = 96.283 (2)°
*V* = 1712.31 (8) Å^3^
*Z* = 4
Cu *K*α radiation
μ = 3.58 mm^−1^
*T* = 296 K
0.26 × 0.25 × 0.11 mm

#### Data collection

Bruker APEXII CCD diffractometer
Absorption correction: multi-scan (*SADABS*; Bruker, 2005 ▶) *T*_min_ = 0.415, *T*_max_ = 0.674
12489 measured reflections
3040 independent reflections
2521 reflections with *I* > 2σ(*I*)
*R*_int_ = 0.042

#### Refinement

*R*[*F* ^2^ > 2σ(*F* ^2^)] = 0.039
*wR*(*F* ^2^) = 0.112
*S* = 1.05
3040 reflections
280 parameters
All H-atom parameters refined
Δρ_max_ = 0.28 e Å^−3^
Δρ_min_ = −0.29 e Å^−3^

### 

Data collection: *APEX2* (Bruker, 2005 ▶); cell refinement: *SAINT* (Bruker, 2005 ▶); data reduction: *SAINT*; program(s) used to solve structure: *SHELXS97* (Sheldrick, 2008 ▶); program(s) used to refine structure: *SHELXL97* (Sheldrick, 2008 ▶); molecular graphics: *ORTEP-3* (Farrugia, 1997 ▶); software used to prepare material for publication: *WinGX* (Farrugia, 1999 ▶).

## Supplementary Material

Crystal structure: contains datablocks I, global. DOI: 10.1107/S160053681003919X/fj2343sup1.cif

Structure factors: contains datablocks I. DOI: 10.1107/S160053681003919X/fj2343Isup2.hkl

Additional supplementary materials:  crystallographic information; 3D view; checkCIF report

## Figures and Tables

**Table 1:** Hydrogen-bond geometry (Å, °)

*D*—H⋯*A*	*D*—H	H⋯*A*	*D*⋯*A*	*D*—H⋯*A*
C12—H122⋯O10	0.97 (2)	2.41 (2)	2.848 (2)	106.5 (17)
C26—H261⋯N6	0.90 (2)	2.36 (2)	2.918 (3)	120.3 (19)
O10—H101⋯O11^i^	0.85 (2)	1.80 (2)	2.6418 (18)	172 (3)
C33—H331⋯Cl27^ii^	0.93 (4)	2.81 (4)	3.534 (2)	135 (3)

Symmetry codes: (i) 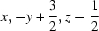; (ii) 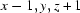.

**Fig. 1.**
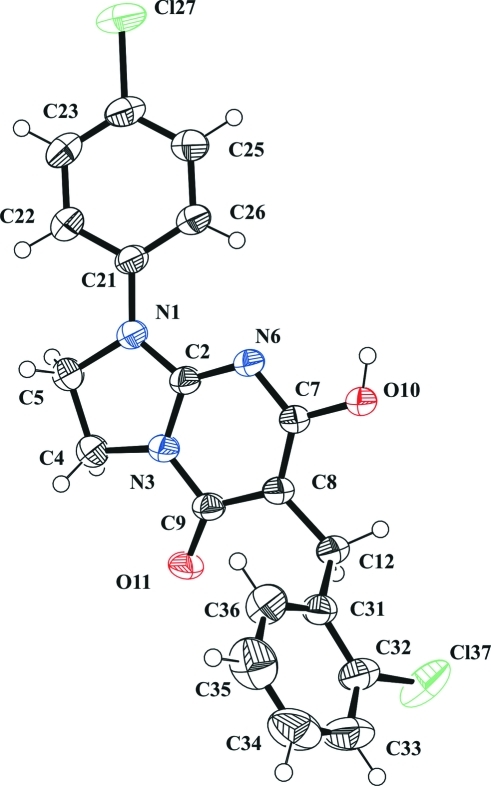
The molecular structure of (I), with atom labels and 50% probability displacement ellipsoids for non-H atoms.

**Fig. 2.**
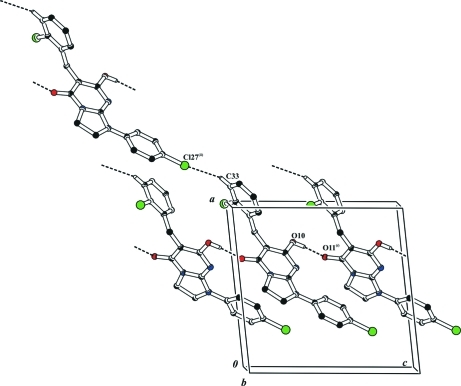
A view of the molecular packing in (I). Dashed lines indicate O—H···O hydrogen bonds and weak C—H···Cl intermolecular interactions.

## supplementary crystallographic information

### Comment

Dioxo derivatives of fused imidazoline ring systems were found to have significant analgesic, opioid-like action but without typical narcotic analgesic side effects (Matosiuk, Fidecka, Antkiewicz-Michaluk, Dybała *et al.*., 2002; Matosiuk, Fidecka, Antkiewicz-Michaluk, Lipkowski *et al.*, 2002). The X-ray analysis of the title compound, (I), was performed in order to confirm the synthesis pathway and identification of its tautomeric form in the solid state. The bond lengths, angles and planarity of the rings in the bicyclic imidazopyrimidine part of (I) are very similar to those observed in previously reported crystal structure of 6-(benzyl)-7-hydroxy-1-(2-methoxyphenyl)-2,3-dihydro-1*H*7*H-* imidazo[1,2-*a*]-pyrimidin-5-one (Wysocki *et al.* (2006). In the crystalline state, the molecule exists as 7-hydroxy-5-oxo tautomer, as evidenced by the C7—O10 [1.330 (2) Å], C9—O11 [1.242 (2) Å], C7—N6 [1.361 (2) Å], C2—N6 [1.305 (2) Å], C9—N3 [1.391 (2) Å] C2—N3 [1.358 (2) Å] bond lengths and the position of the H atom in the vicinity of O10 in difference electron-density map. The dihedral angles between the fused imidazopyrimidine and aromatic chlopophenyl and chlobobenzyl rings are 14.2 (1) and 70.7 (1)°, respectively. This conformation is influenced by the intramolecular C12—H122···O10 and C26—H261···N6 hydrogen bonds giving nearly co-planar five-ring fused system. In the crystal structure, strong intermolecular O10—H101···O11 resonance-assisted hydrogen bond (Gilli *et al.*, 1989) links the molecules related by *c*-glide plane into chains along the *c* axis. Additionaly, molecules are joined in molecular chains parallel to [101] direction by a C33—H331···Cl27 hydrogen bond. Moreover, the guanidine *π*-electron system and phenyl ring, belonging to inversion-related molecules overlap with the shortest intermolecular contact C2···C23^iii^ of 3.270 (3) and the angle between overlapping planes of 13.33 (11)° characteristic for *π*-*π* interactions [(iii) = 1 - *x*, 1 - *y*, 1 - *z*].

### Experimental

The title compound, C_19_H_15_Cl_2_N_3_O_2_ (I), was obtained by one-step cyclocondensation of 1-(4-chlorophenyl)-2-aminoimidazoline-2 with diethyl (2-chlorobenzyl)malonate under basic (sodium methoxide) conditions (Rządkowska *et al.*, 2004). Crystals suitable for X-ray diffraction analysis were grown by slow evaporation of a propan-2-ol solution.

### Refinement

All H atoms were located in difference Fourier maps and refined freely with *U*_iso_(H) values of 1.5*U*_eq_(N,*C*, O).

### Crystal data

**Table:**

C_19_H_15_Cl_2_N_3_O_2_	*F*(000) = 800
*M**_r_* = 388.24	*D*_x_ = 1.506 Mg m^−^^3^
Monoclinic, *P*2_1_/*c*	Cu *K*α radiation, λ = 1.54178 Å
Hall symbol: -P 2ybc	Cell parameters from 3410 reflections
*a* = 11.4521 (3) Å	θ = 3.9–66.7°
*b* = 12.8287 (4) Å	µ = 3.58 mm^−^^1^
*c* = 11.7255 (3) Å	*T* = 296 K
β = 96.283 (2)°	Block, colourless
*V* = 1712.31 (8) Å^3^	0.26 × 0.25 × 0.11 mm
*Z* = 4	

### Data collection

**Table:**

Bruker APEXII CCD diffractometer	3040 independent reflections
Radiation source: fine-focus sealed tube	2521 reflections with *I* > 2σ(*I*)
graphite	*R*_int_ = 0.042
φ and ω scans	θ_max_ = 67.8°, θ_min_ = 3.9°
Absorption correction: multi-scan (*SADABS*; Bruker, 2005)	*h* = −13→12
*T*_min_ = 0.415, *T*_max_ = 0.674	*k* = −15→8
12489 measured reflections	*l* = −13→13

### Refinement

**Table:**

Refinement on *F*^2^	Primary atom site location: structure-invariant direct methods
Least-squares matrix: full	Secondary atom site location: difference Fourier map
*R*[*F*^2^ > 2σ(*F*^2^)] = 0.039	Hydrogen site location: difference Fourier map
*wR*(*F*^2^) = 0.112	All H-atom parameters refined
*S* = 1.05	*w* = 1/[σ^2^(*F*_o_^2^) + (0.0566*P*)^2^ + 0.4965*P*] where *P* = (*F*_o_^2^ + 2*F*_c_^2^)/3
3040 reflections	(Δ/σ)_max_ < 0.001
280 parameters	Δρ_max_ = 0.28 e Å^−^^3^
0 restraints	Δρ_min_ = −0.29 e Å^−^^3^

### Special details

**Table:**

Geometry. All e.s.d.'s (except the e.s.d. in the dihedral angle between two l.s. planes) are estimated using the full covariance matrix. The cell e.s.d.'s are taken into account individually in the estimation of e.s.d.'s in distances, angles and torsion angles; correlations between e.s.d.'s in cell parameters are only used when they are defined by crystal symmetry. An approximate (isotropic) treatment of cell e.s.d.'s is used for estimating e.s.d.'s involving l.s. planes. Weighted least-squares planes through the starred atoms (Nardelli, Musatti, Domiano & Andreetti Ric.Sci.(1965),15(II—A),807). Equation of the plane: m1**X*+m2*Y+m3**Z*=dPlane 1 m1 = -0.57842(0.00033) m2 = -0.67971(0.00033) m3 = -0.45102(0.00053) D = -11.68389(0.00244) Atom d s d/s (d/s)**2 N1 * -0.0460 0.0016 - 28.384 805.647 C2 * 0.0029 0.0018 1.594 2.541 N3 * 0.0198 0.0015 13.215 174.625 C4 * -0.0357 0.0023 - 15.771 248.720 C5 * 0.0819 0.0023 36.135 1305.707 N6 * 0.0038 0.0015 2.558 6.543 C7 * 0.0139 0.0017 7.983 63.731 C8 * 0.0007 0.0018 0.384 0.147 C9 * -0.0243 0.0018 - 13.656 186.495 ============ Sum((d/s)**2) for starred atoms 2794.157 Chi-squared at 95% for 6 degrees of freedom: 12.60 The group of atoms deviates significantly from planarityPlane 2 m1 = -0.71141(0.00065) m2 = -0.48107(0.00093) m3 = -0.51231(0.00073) D = -11.40891(0.00306) Atom d s d/s (d/s)**2 C21 * 0.0042 0.0018 2.264 5.124 C22 * -0.0048 0.0022 - 2.159 4.661 C23 * -0.0012 0.0023 - 0.496 0.246 C24 * 0.0051 0.0021 2.408 5.797 C25 * -0.0050 0.0024 - 2.037 4.149 C26 * -0.0018 0.0023 - 0.782 0.611 ============ Sum((d/s)**2) for starred atoms 20.589 Chi-squared at 95% for 3 degrees of freedom: 7.81 The group of atoms deviates significantly from planarityPlane 3 m1 = -0.47124(0.00082) m2 = 0.42681(0.00103) m3 = -0.77186(0.00062) D = -3.90575(0.01406) Atom d s d/s (d/s)**2 C31 * -0.0026 0.0019 - 1.391 1.935 C32 * 0.0016 0.0021 0.757 0.573 C33 * 0.0031 0.0027 1.149 1.321 C34 * -0.0079 0.0031 - 2.556 6.533 C35 * 0.0042 0.0031 1.328 1.764 C36 * 0.0021 0.0024 0.859 0.738 ============ Sum((d/s)**2) for starred atoms 12.865 Chi-squared at 95% for 3 degrees of freedom: 7.81 The group of atoms deviates significantly from planarityDihedral angles formed by LSQ-planes Plane - plane angle (s.u.) angle (s.u.) 1 2 14.18 (0.06) 165.82 (0.06) 1 3 70.69 (0.06) 109.31 (0.06) 2 3 58.31 (0.06) 121.69 (0.06)
Refinement. Refinement of *F*^2^ against ALL reflections. The weighted *R*-factor *wR* and goodness of fit *S* are based on *F*^2^, conventional *R*-factors *R* are based on *F*, with *F* set to zero for negative *F*^2^. The threshold expression of *F*^2^ > σ(*F*^2^) is used only for calculating *R*-factors(gt) *etc*. and is not relevant to the choice of reflections for refinement. *R*-factors based on *F*^2^ are statistically about twice as large as those based on *F*, and *R*- factors based on ALL data will be even larger.

### Fractional atomic coordinates and isotropic or equivalent isotropic displacement parameters (Å^2^)

**Table:**

	*x*	*y*	*z*	*U*_iso_*/*U*_eq_	
N1	0.54690 (14)	0.58844 (13)	0.65922 (13)	0.0405 (4)	
C2	0.45149 (16)	0.63750 (14)	0.69364 (15)	0.0345 (4)	
N3	0.42905 (13)	0.59910 (12)	0.79699 (12)	0.0368 (4)	
C4	0.5131 (2)	0.51928 (18)	0.84006 (18)	0.0470 (5)	
H41	0.560 (2)	0.547 (2)	0.910 (2)	0.070*	
H42	0.473 (2)	0.454 (2)	0.860 (2)	0.070*	
C5	0.5851 (2)	0.50339 (18)	0.73916 (18)	0.0472 (5)	
H51	0.670 (3)	0.511 (2)	0.762 (2)	0.071*	
H52	0.567 (2)	0.430 (2)	0.703 (2)	0.071*	
N6	0.39106 (13)	0.71171 (12)	0.63876 (12)	0.0362 (4)	
C7	0.30136 (15)	0.75057 (14)	0.69309 (14)	0.0329 (4)	
C8	0.27243 (16)	0.71925 (14)	0.79896 (15)	0.0349 (4)	
C9	0.34131 (16)	0.63956 (14)	0.85735 (14)	0.0346 (4)	
O10	0.24079 (12)	0.82712 (10)	0.63775 (11)	0.0402 (3)	
H101	0.268 (2)	0.844 (2)	0.576 (2)	0.060*	
O11	0.33241 (12)	0.60419 (10)	0.95466 (10)	0.0423 (3)	
C12	0.17750 (17)	0.77055 (15)	0.85764 (17)	0.0390 (4)	
H121	0.213 (2)	0.7977 (18)	0.933 (2)	0.059*	
H122	0.150 (2)	0.832 (2)	0.814 (2)	0.059*	
C21	0.59787 (16)	0.60289 (15)	0.55615 (15)	0.0384 (4)	
C22	0.67539 (19)	0.52838 (18)	0.52397 (19)	0.0500 (5)	
H221	0.691 (2)	0.465 (2)	0.568 (2)	0.075*	
C23	0.7284 (2)	0.53957 (19)	0.4242 (2)	0.0534 (6)	
H231	0.777 (3)	0.490 (2)	0.404 (2)	0.080*	
C24	0.70459 (18)	0.62511 (18)	0.35695 (18)	0.0499 (5)	
C25	0.6293 (2)	0.7008 (2)	0.3878 (2)	0.0568 (6)	
H251	0.617 (3)	0.764 (2)	0.342 (2)	0.085*	
C26	0.5754 (2)	0.69008 (19)	0.48719 (19)	0.0511 (5)	
H261	0.529 (2)	0.742 (2)	0.507 (2)	0.077*	
Cl27	0.77155 (6)	0.63967 (6)	0.23211 (5)	0.0784 (2)	
C31	0.07284 (16)	0.70368 (15)	0.87807 (16)	0.0389 (4)	
C32	−0.00662 (18)	0.73742 (19)	0.95069 (17)	0.0489 (5)	
C33	−0.1022 (2)	0.6786 (3)	0.9736 (2)	0.0669 (7)	
H331	−0.152 (3)	0.706 (3)	1.024 (3)	0.100*	
C34	−0.1203 (2)	0.5828 (3)	0.9241 (3)	0.0747 (8)	
H341	−0.183 (3)	0.540 (3)	0.942 (3)	0.112*	
C35	−0.0442 (2)	0.5470 (2)	0.8503 (3)	0.0742 (8)	
H351	−0.054 (3)	0.479 (3)	0.814 (3)	0.111*	
C36	0.0516 (2)	0.60699 (18)	0.8281 (2)	0.0541 (5)	
H361	0.103 (3)	0.581 (2)	0.779 (2)	0.081*	
Cl37	0.01144 (6)	0.85933 (6)	1.01526 (7)	0.0825 (3)	

### Atomic displacement parameters (Å^2^)

**Table:**

	*U*^11^	*U*^22^	*U*^33^	*U*^12^	*U*^13^	*U*^23^
N1	0.0425 (8)	0.0461 (9)	0.0342 (8)	0.0134 (7)	0.0110 (7)	0.0067 (7)
C2	0.0374 (9)	0.0386 (9)	0.0281 (8)	0.0033 (8)	0.0064 (7)	0.0001 (7)
N3	0.0419 (8)	0.0412 (8)	0.0283 (7)	0.0055 (7)	0.0082 (6)	0.0041 (6)
C4	0.0524 (12)	0.0506 (12)	0.0387 (10)	0.0142 (10)	0.0088 (9)	0.0098 (10)
C5	0.0523 (12)	0.0496 (12)	0.0405 (11)	0.0157 (10)	0.0089 (9)	0.0103 (9)
N6	0.0395 (8)	0.0411 (8)	0.0296 (7)	0.0070 (7)	0.0107 (6)	0.0026 (6)
C7	0.0364 (9)	0.0338 (9)	0.0293 (8)	0.0012 (7)	0.0071 (7)	0.0003 (7)
C8	0.0391 (9)	0.0364 (9)	0.0305 (9)	0.0004 (8)	0.0100 (7)	0.0008 (8)
C9	0.0397 (9)	0.0368 (9)	0.0284 (9)	−0.0036 (8)	0.0082 (7)	−0.0026 (7)
O10	0.0456 (7)	0.0434 (7)	0.0335 (7)	0.0109 (6)	0.0129 (6)	0.0068 (6)
O11	0.0570 (8)	0.0452 (7)	0.0265 (6)	0.0006 (6)	0.0127 (6)	0.0035 (6)
C12	0.0436 (10)	0.0387 (10)	0.0370 (10)	0.0016 (8)	0.0145 (8)	−0.0005 (9)
C21	0.0363 (9)	0.0467 (10)	0.0334 (9)	0.0055 (8)	0.0092 (7)	−0.0008 (8)
C22	0.0525 (12)	0.0496 (12)	0.0506 (12)	0.0134 (10)	0.0173 (10)	0.0013 (10)
C23	0.0525 (12)	0.0555 (13)	0.0557 (13)	0.0110 (11)	0.0212 (10)	−0.0078 (11)
C24	0.0458 (11)	0.0655 (14)	0.0408 (11)	0.0029 (10)	0.0162 (9)	−0.0037 (10)
C25	0.0606 (14)	0.0656 (14)	0.0476 (12)	0.0159 (12)	0.0211 (10)	0.0134 (11)
C26	0.0538 (12)	0.0584 (13)	0.0447 (11)	0.0178 (11)	0.0210 (9)	0.0086 (10)
Cl27	0.0858 (5)	0.1015 (5)	0.0555 (4)	0.0106 (4)	0.0425 (3)	0.0026 (3)
C31	0.0380 (9)	0.0458 (10)	0.0335 (9)	0.0007 (8)	0.0067 (7)	0.0036 (8)
C32	0.0413 (10)	0.0673 (14)	0.0395 (10)	0.0036 (10)	0.0105 (8)	0.0005 (10)
C33	0.0432 (12)	0.104 (2)	0.0555 (14)	−0.0044 (13)	0.0165 (10)	0.0133 (15)
C34	0.0489 (13)	0.092 (2)	0.0832 (19)	−0.0194 (14)	0.0082 (13)	0.0247 (17)
C35	0.0642 (16)	0.0622 (15)	0.094 (2)	−0.0200 (13)	−0.0012 (15)	−0.0006 (15)
C36	0.0501 (12)	0.0532 (13)	0.0597 (14)	−0.0049 (10)	0.0091 (10)	−0.0086 (11)
Cl37	0.0628 (4)	0.0959 (5)	0.0929 (5)	0.0075 (3)	0.0269 (3)	−0.0442 (4)

### Geometric parameters (Å, °)

**Table:**

N1—C2	1.360 (2)	C21—C22	1.385 (3)
N1—C21	1.411 (2)	C21—C26	1.388 (3)
N1—C5	1.473 (2)	C22—C23	1.383 (3)
C2—N6	1.305 (2)	C22—H221	0.97 (3)
C2—N3	1.358 (2)	C23—C24	1.362 (3)
N3—C9	1.391 (2)	C23—H231	0.90 (3)
N3—C4	1.458 (2)	C24—C25	1.373 (3)
C4—C5	1.528 (3)	C24—Cl27	1.735 (2)
C4—H41	0.99 (3)	C25—C26	1.384 (3)
C4—H42	0.99 (3)	C25—H251	0.97 (3)
C5—H51	0.99 (3)	C26—H261	0.90 (3)
C5—H52	1.04 (3)	C31—C36	1.382 (3)
N6—C7	1.361 (2)	C31—C32	1.382 (3)
C7—O10	1.330 (2)	C32—C33	1.381 (3)
C7—C8	1.379 (2)	C32—Cl37	1.740 (2)
C8—C9	1.421 (3)	C33—C34	1.364 (4)
C8—C12	1.501 (2)	C33—H331	0.93 (3)
C9—O11	1.242 (2)	C34—C35	1.374 (4)
O10—H101	0.85 (3)	C34—H341	0.95 (4)
C12—C31	1.514 (3)	C35—C36	1.388 (3)
C12—H121	1.00 (3)	C35—H351	0.97 (4)
C12—H122	0.97 (3)	C36—H361	0.93 (3)
			
C2—N1—C21	127.93 (15)	H121—C12—H122	105.4 (19)
C2—N1—C5	110.25 (15)	C22—C21—C26	118.77 (18)
C21—N1—C5	121.37 (15)	C22—C21—N1	118.71 (18)
N6—C2—N3	124.25 (16)	C26—C21—N1	122.50 (17)
N6—C2—N1	126.25 (16)	C23—C22—C21	120.8 (2)
N3—C2—N1	109.48 (15)	C23—C22—H221	117.5 (16)
C2—N3—C9	122.42 (15)	C21—C22—H221	121.6 (16)
C2—N3—C4	112.43 (15)	C24—C23—C22	119.7 (2)
C9—N3—C4	124.92 (15)	C24—C23—H231	120.6 (19)
N3—C4—C5	102.55 (15)	C22—C23—H231	119.7 (19)
N3—C4—H41	108.5 (15)	C23—C24—C25	120.6 (2)
C5—C4—H41	113.2 (15)	C23—C24—Cl27	119.78 (17)
N3—C4—H42	111.4 (15)	C25—C24—Cl27	119.62 (18)
C5—C4—H42	112.2 (15)	C24—C25—C26	120.2 (2)
H41—C4—H42	109 (2)	C24—C25—H251	120.1 (17)
N1—C5—C4	104.26 (16)	C26—C25—H251	119.6 (17)
N1—C5—H51	108.7 (16)	C25—C26—C21	120.0 (2)
C4—C5—H51	112.1 (16)	C25—C26—H261	118.3 (18)
N1—C5—H52	111.8 (14)	C21—C26—H261	121.7 (18)
C4—C5—H52	109.6 (14)	C36—C31—C32	116.41 (19)
H51—C5—H52	110 (2)	C36—C31—C12	123.18 (17)
C2—N6—C7	115.03 (15)	C32—C31—C12	120.40 (18)
O10—C7—N6	114.97 (15)	C33—C32—C31	122.6 (2)
O10—C7—C8	119.37 (16)	C33—C32—Cl37	117.77 (19)
N6—C7—C8	125.63 (16)	C31—C32—Cl37	119.65 (16)
C7—C8—C9	117.90 (16)	C34—C33—C32	119.8 (2)
C7—C8—C12	122.85 (17)	C34—C33—H331	122 (2)
C9—C8—C12	119.12 (15)	C32—C33—H331	118 (2)
O11—C9—N3	117.86 (16)	C33—C34—C35	119.5 (2)
O11—C9—C8	127.47 (16)	C33—C34—H341	121 (2)
N3—C9—C8	114.66 (15)	C35—C34—H341	120 (2)
C7—O10—H101	113.1 (17)	C34—C35—C36	120.1 (3)
C8—C12—C31	116.73 (16)	C34—C35—H351	122 (2)
C8—C12—H121	108.5 (14)	C36—C35—H351	118 (2)
C31—C12—H121	107.9 (14)	C31—C36—C35	121.7 (2)
C8—C12—H122	109.0 (14)	C31—C36—H361	119.5 (19)
C31—C12—H122	108.6 (14)	C35—C36—H361	118.8 (19)
			
C21—N1—C2—N6	−3.2 (3)	C7—C8—C12—C31	116.6 (2)
C5—N1—C2—N6	−175.39 (19)	C9—C8—C12—C31	−67.8 (2)
C21—N1—C2—N3	178.28 (17)	C2—N1—C21—C22	−164.0 (2)
C5—N1—C2—N3	6.0 (2)	C5—N1—C21—C22	7.5 (3)
N6—C2—N3—C9	−3.1 (3)	C2—N1—C21—C26	17.6 (3)
N1—C2—N3—C9	175.46 (16)	C5—N1—C21—C26	−171.0 (2)
N6—C2—N3—C4	−177.88 (19)	C26—C21—C22—C23	−0.9 (3)
N1—C2—N3—C4	0.7 (2)	N1—C21—C22—C23	−179.4 (2)
C2—N3—C4—C5	−6.7 (2)	C21—C22—C23—C24	0.3 (4)
C9—N3—C4—C5	178.73 (18)	C22—C23—C24—C25	0.6 (4)
C2—N1—C5—C4	−9.9 (2)	C22—C23—C24—Cl27	179.68 (18)
C21—N1—C5—C4	177.28 (18)	C23—C24—C25—C26	−0.9 (4)
N3—C4—C5—N1	9.5 (2)	Cl27—C24—C25—C26	180.0 (2)
N3—C2—N6—C7	0.4 (3)	C24—C25—C26—C21	0.4 (4)
N1—C2—N6—C7	−177.92 (18)	C22—C21—C26—C25	0.6 (3)
C2—N6—C7—O10	179.17 (15)	N1—C21—C26—C25	179.0 (2)
C2—N6—C7—C8	1.1 (3)	C8—C12—C31—C36	−12.0 (3)
O10—C7—C8—C9	−178.03 (16)	C8—C12—C31—C32	167.16 (18)
N6—C7—C8—C9	−0.1 (3)	C36—C31—C32—C33	0.3 (3)
O10—C7—C8—C12	−2.3 (3)	C12—C31—C32—C33	−178.9 (2)
N6—C7—C8—C12	175.65 (17)	C36—C31—C32—Cl37	−178.73 (17)
C2—N3—C9—O11	−175.20 (17)	C12—C31—C32—Cl37	2.1 (3)
C4—N3—C9—O11	−1.1 (3)	C31—C32—C33—C34	0.4 (4)
C2—N3—C9—C8	3.9 (2)	Cl37—C32—C33—C34	179.5 (2)
C4—N3—C9—C8	178.01 (19)	C32—C33—C34—C35	−1.2 (4)
C7—C8—C9—O11	176.67 (18)	C33—C34—C35—C36	1.2 (4)
C12—C8—C9—O11	0.8 (3)	C32—C31—C36—C35	−0.3 (3)
C7—C8—C9—N3	−2.4 (2)	C12—C31—C36—C35	178.9 (2)
C12—C8—C9—N3	−178.25 (16)	C34—C35—C36—C31	−0.5 (4)

### Hydrogen-bond geometry (Å, °)

**Table:**

*D*—H···*A*	*D*—H	H···*A*	*D*···*A*	*D*—H···*A*
C12—H122···O10	0.97 (2)	2.41 (2)	2.848 (2)	106.5 (17)
C26—H261···N6	0.90 (2)	2.36 (2)	2.918 (3)	120.3 (19)
O10—H101···O11^i^	0.85 (2)	1.80 (2)	2.6418 (18)	172 (3)
C33—H331···Cl27^ii^	0.93 (4)	2.81 (4)	3.534 (2)	135 (3)

Symmetry codes: (i) *x*, −*y*+3/2, *z*−1/2; (ii) *x*−1, *y*, *z*+1.
